# Health coaching for people with long-term conditions and multimorbidity: a mixed methods prospective service evaluation of Structured Agenda-free Coaching Conversations (StACC) in UK primary care

**DOI:** 10.1186/s12889-025-23535-0

**Published:** 2025-07-03

**Authors:** Kate Henry, Austen El-Osta, Kathleen Leedham-Green

**Affiliations:** 1 Know Your Own Health Limited, 27 Mortimer Street, London, W1T 3BL UK; 2 Department of Primary Care and Public Health, Self-Care Academic Research Unit (SCARU), School of Public Health, Imperial College, London, UK; 3 Faculty of Medicine, Imperial College London, London, UK

**Keywords:** Health education, Self-management, Patient education, Multiple chronic conditions, Chronic illness

## Abstract

**Background:**

Enhanced patient engagement has been linked to health service sustainability. The Structured Agenda-free Coaching Conversation (StACC) model aims to support people with one or more long-term conditions through a strengths-based approach: emphasising patient ownership and engagement rather than specific health-related knowledge, skills or lifestyle-related goals. The intervention consists of five or six personalised one-to-one coaching sessions with a non-clinical coach. This evaluation explored the impacts of StACC on patient activation, self-management capabilities, patient experience and health service use.

**Methods:**

A prospective service evaluation was conducted across seven UK general practices between 2015 and 2018. Patient-reported outcome measures were the Patient Activation Measure (PAM-13) or the Health Education Impact Questionnaire (heiQ). Patient stories and feedback were thematically summarised. Health service usage (encounters) was explored for a small subgroup 12 months before and after the intervention.

**Results:**

A total of 1,031 participants were onboarded, and 630 (61.1%) completed the intervention. Of those, 575 (91.3%) provided paired evaluative data for either PAM-13 (*n* = 110) or heiQ (*n* = 465), 586 completed feedback questionnaires (93.0%), and 376 (59.7%) agreed to share their individual stories. Complete health service data were provided for a small subgroup (*n* = 58, 9.2% of completers). The pre-intervention mean PAM-13 score was 57.1 (SD = 13.6, 95% CI = 54.5–59.7). This increased to 70.8 (SD = 14.7, 95% CI = 68.1–73.6) post-intervention (*p* < 0.0001). There were improvements across all eight domains of heiQ (effect sizes between 0.36 and 0.66). Health service data suggested a sustained impact. Participants who were previously disengaged reported taking action and gaining more control. Of the 586 feedback questionnaires, 567 (96.8%) were positive.

**Conclusions:**

Personalised health coaching delivered through the StACC model increased patient activation (quantitative evidence) and improved self-management behaviours (qualitative evidence) for participants. This is supported by exploratory health service data. As this was an uncontrolled service evaluation, and the appointment data subgroup was small, follow-up efficacy and economic studies are warranted.

**Supplementary Information:**

The online version contains supplementary material available at 10.1186/s12889-025-23535-0.

## Background

Supporting people with long-term conditions and multimorbidity to manage their health and wellbeing effectively is one of the cornerstones of a sustainable healthcare system [1–6]. The Chronic Care Model [7] and the related House of Care model [8] both refer to ‘engaged, informed patients’ as necessary components [9]. Without first ensuring that patients are engaged and taking ownership of their health and wellbeing, they will not necessarily be able to play their part in these models.

This is also relevant to the Wanless Report of 2002 [2]. Its optimistic ‘fully engaged’ scenario for the UK’s National Health Service relies on patient and population engagement and ownership. This point was reiterated in the Wanless review of 2012 [10] which stated that: “*Major features of the ‘fully engaged’ scenario include a massive improvement in the public’s engagement in their own health …[and]… a dramatic improvement in public health… as people take ownership of their own health.”*

Supported self-management programmes are often designed around a single condition, such as diabetes or COPD, where participants are expected to acquire pre-specified knowledge and skills. Where self-management programmes use coaching, the focus is often on achieving pre-specified behavioural outcomes such as smoking cessation or weight loss. Even where the coaching topic is negotiated, as in the T-GROW model [11], the focus tends to be around defining and achieving a behavioural outcome. Although knowledge, skills and behaviours are important, there are also underlying factors that impact on effective self-management including social and psychological determinants [12]. Pertinently, one’s psychological approach is potentially modifiable [13].

Patient activation as a psychological construct is closely related to self-efficacy [14] which is a person’s confidence in their ability to perform tasks and problem-solve and whether they see themself as having an active role in their own outcomes. An observational study by Greene et al. demonstrated correlations between patient activation and better health outcomes, improved wellbeing, better self-management and a reduced need for acute healthcare [15]. Despite numerous other studies showcasing the connection between patient activation and health outcomes [16–18], the extent to which patient activation can be modified through a direct intervention has not been firmly established, nor whether this leads to measurable improvements in outcomes and a reduced reliance on clinical services.

### The StACC intervention

The Structured Agenda-Free Coaching Conversation (StACC) model is a form of coaching that emerged as a result of the Self-Management Programme within the Health Foundation’s Co-Creating Health initiative [19, 20] and the coaching conversation steps used in that programme. It combines elements of the Expert Patient Programme [3], Stanford University’s Chronic Disease Self-Management Programme [21, 22] and Motivational Interviewing [23]. Whereas StACC shares generic coaching skills and steps with other supported self-management interventions and coaching approaches, the difference is in how it focuses on patient ownership and engagement, patient activation and internalisation of confidence-building and problem-solving. Crucially, the primary aim of StACC is to increase patient activation and thereby promote self-management rather than the obverse, primarily supporting self-management and thereby increasing patient activation. The premise is that people who are more ‘activated’ will build their own knowledge, skills and confidence to self-manage effectively and be able to do so on a more sustainable basis.

**Fig. 1:**
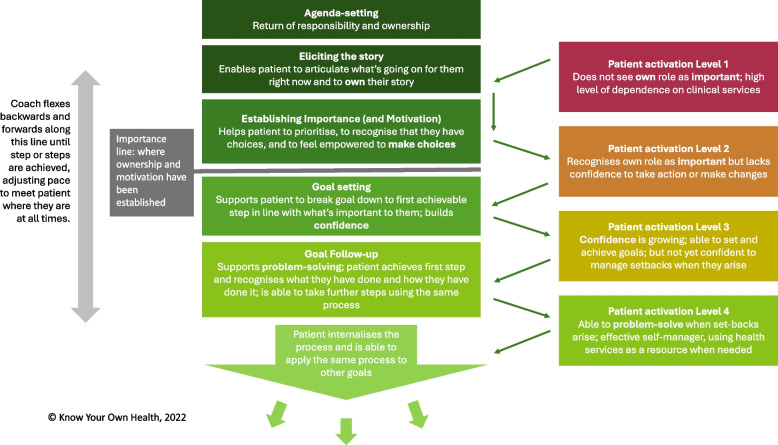
Structured Agenda-Free Coaching Conversations (StACC) model, mapped to Patient Activation (PAM) level

The StACC intervention consists of a short series of one-to-one coaching sessions, delivered by trained and supported coaches who use a defined set of skills in a structured way (Fig. 1). The approach is not mono-disease, -issue, or -behaviour specific and is therefore suitable for people with one or more long-term conditions or issues. The StACC model prioritises personal ownership and engagement and builds on the individual’s intrinsic motivation to improve their health and wellbeing. Once ownership and engagement are established, the coach continues to guide the individual with a progressively narrow focus around what is important to them and what steps they need to take to achieve it. The process follows a ‘journey arc’, with coaches working backwards and forwards as necessary through the steps until a meaningful goal, or step(s) towards it, is achieved. This structured approach keeps the discussion on topic whilst also allowing the coach to work flexibly, adjusting the pace to suit the individual and meet them where they are at all times, whatever their initial activation level.

StACC involves five coaching sessions, usually over 5–10 weeks, with an optional follow-up a month later. The number of sessions and the timeframe are adjusted according to need. The first session lasts an hour, and subsequent sessions last 45 min. Fidelity to the StACC model is supported through training and supervision. Initial training lasts two days, followed by six individual supervision sessions alongside the coach’s initial caseload and monthly group skills-development sessions. Thereafter, each coach has one-to-one supervisions with a trainer-level supervisor in the StACC approach every 1–2 months with additional support as needed (until the coach reaches trainer level themselves and moves to peer group support and supervision). All patient contact is logged on a purpose-built online platform supporting case-load management, reporting and evaluation.

In this implementation, all sessions were delivered face-to-face at the participant’s GP practice or a local venue. Up to 10 part-time coaches worked concurrently, each typically seeing 5–6 patients daily. The coaches all had a background involving listening skills, such as counselling, mentoring, or working with people in a supportive role.

## Methods

### Aim

To evaluate StACC's impacts on patient activation, self-management capabilities, patient experience, and health service use.

### Ethics

This study was classed as a service evaluation and exempted from research ethics processes as confirmed by the NHS Health Research Authority [24]. Evaluation ethics were reviewed by the NHS commissioning organisation, including consent processes, confidentiality, and data protection. All questionnaires and demographic questions were optional, and participants were advised that they could withdraw their consent at any time. All potentially identifying data were removed. Informed written consent was obtained for the use of individual patient stories.

### Design

This was a mixed-methods prospective service evaluation comparing validated patient-reported outcome measures (either PAM-13 or heiQ, see below) before and after the StACC intervention, triangulated with practice-reported health service usage for a small subset (appointment and admissions data) and qualitative data (feedback comments and patient stories). The primary evaluation outcome measure was changed from heiQ to PAM-13 for the final six months on request of the NHS commissioning organisation in response to local evaluative policy. However, the intervention remained the same, allowing outcomes for the different measures to be compared.

### Setting

The evaluation was conducted between April 2015 and September 2018 in one NHS England commissioning area. The StACC intervention was initiated at three primary care practices and expanded to four additional practices in the second half of the evaluation period.

### Timeline

Matched heiQ data were collected at baseline and on completion between April 2015 and March 2018. Matched PAM-13 data were collected at baseline and on completion between April 2018 and September 2018. Demographic data were recorded on registration. Experience and feedback data were recorded on completion. Monthly health service usage data were requested in August 2017. These were collated for 12 months before and after the final coaching session; therefore, only included a small subset of participants who completed before August 2016 from the original three primary care sites. All health service usage records with complete data 12 months before and after the final intervention session were included in the analysis.

### Participants

Eligibility criteria were adults registered at participating NHS practices with one or more long-term conditions who may be struggling to manage their (physical or mental) health condition(s) or its impacts (e.g., isolation, fatigue, ability to work or to fully engage with life). Eligible patients were identified from practice lists and invited by letter, or GPs could refer directly. Exclusion criteria were dementia or receiving active psychiatric, cancer, or end-of-life care. Those responding to the invitation were contacted by a trained coach who described the service and asked if they would like to participate.

### Measures

PAM-13 and heiQ were licensed for use by the NHS commissioning organisation (see ‘Funding’ statement) and directly with the licensor.PAM and heiQ data were collected using paper forms, entered on the online platform by their coach, and collated by the programme lead (KH) on behalf of the NHS commissioning organisation.

#### Health Education Impact Questionnaire (heiQ)

The Health Education Impact Questionnaire (heiQ) is a 40-question instrument with good internal reliability (Cronbach’s alpha 0.70–0.89) that has been validated in multiple populations and contexts [25]. It measures the impacts of self-management programmes across eight constructs: (i) health-directed behaviour, (ii) positive and active engagement in life, (iii) self-monitoring and insight, (iv) constructive attitudes and approaches, (v) skill and technique acquisition, (vi) social integration & support, (vii) health services navigation, and (viii) emotional distress [25]. Each question is scored between 1–4, and each domain score is normalised to a maximum of four. Higher scores indicate greater ability to self-manage effectively.

#### Patient Activation Measure (PAM-13)

The Patient Activation Measure (PAM-13) [26, 27] is a 13-item instrument with good-to-excellent reliability (person and item separation reliability 0.81–0.99) that has been validated in multiple contexts [28]. It aims to measure a person's knowledge, skills and confidence in managing their health and wellbeing and is associated with positive self-care, more effective self-management and reduced reliance on acute healthcare [6, 13]. The instrument provides a score from 0–100 and uses proprietary calibration tables to categorise people into four levels of activation, defined as: 1) disengaged and overwhelmed, 2) becoming aware but still struggling; 3) taking action and gaining control; 4) maintaining behaviours and pushing further. Higher PAM scores correlate with positive health behaviours and outcomes [18].

#### Health service usage

Health service usage data were reported for individual participants but not matched to PAM, heiQ or demographic data. These were a monthly count of GP appointments, GP telephone appointments, GP home visits, nurse appointments, health care assistant appointments, out-of-hours visits, hospital admissions and A&E attendances (GP reported).

#### Contextual and qualitative data

All participants who commenced the programme were invited to provide demographic data (age, gender, ethnicity, current diagnoses). Participants who completed the programme were invited to reflect on the programme’s impacts. This was collaboratively recorded with their coach as a ‘patient story’. Participants who did not complete the programme were approached and, where contactable, reasons were recorded. All completers were given feedback questionnaires at the end of the final session to return to the coach or to the practice (9 Likert-scored questions and an optional free text box).

### Reflexivity

Health services evaluation has inherent sources of bias, including differential attrition and researcher biases [29]. KH was the intervention lead, and the health coaches delivering the intervention gathered the evaluative data. Researcher biases were mitigated through mixed methods (each method triangulating the other), validated instruments, and two independent health services researchers (KLG, AEO) who audited the data and its analysis. Attrition is presented transparently and discussed.

### Data analysis

All completed pre- and post-intervention measures were included. Incomplete data were replaced with a neutral value (T2 = T1). A sensitivity analysis was not performed as the missing data were low. Normality of the distribution of the differences between pre- and post-intervention was checked using the Shapiro–Wilk test with histogram visualisation. Statistical analyses were conducted using STATA, version 17 (StataCorp LP, College Station, TX, USA). Statistical significance was assumed at *p* = 0.05. Quantitative data were expressed in numbers (N) and percentages (%).

For heiQ data, the instrument owners (Deakin University) calculated the group effect size according to a published method [22] for skewed data where base and follow-up variances are unequal. They also provided benchmark comparisons based on outcomes from 3,221 Australian participants across diverse supported self-management programmes for people with long-term health conditions.

For the Patient Activation Measures (raw scores), a paired T-test was performed (computing means, standard deviations, and confidence intervals) as the differences were normally distributed. The instrument providers categorised patients into PAM levels. Benchmark PAM data were from 12,270 people with long-term conditions from the Health Foundation Islington Study [16].

For the health service usage data, the distribution of appointments per person was not normal. Therefore, median appointments per person per month and interquartile range are reported, and a Wilcoxon signed-rank test was performed. Results are reported for all available data with unadjusted *p*-values and raw effect sizes. Adjustment for multiple comparisons was not conducted as the results share a causal mechanism (e.g., fewer acute GP appointments are linked to fewer hospital admissions).

A thematic summary of patient stories and feedback was conducted by KH and KLG [30]. This involved KH manually coding excerpts with one or more descriptors (confidence, goals, actions, self-efficacy, impacts, experiences) and KLG summarising the data within each descriptor. KH extracted patient-reported outcomes from consented patient stories. These were inductively grouped into related outcomes and charted according to frequency.

## Results

### Participation

**Fig. 2:**
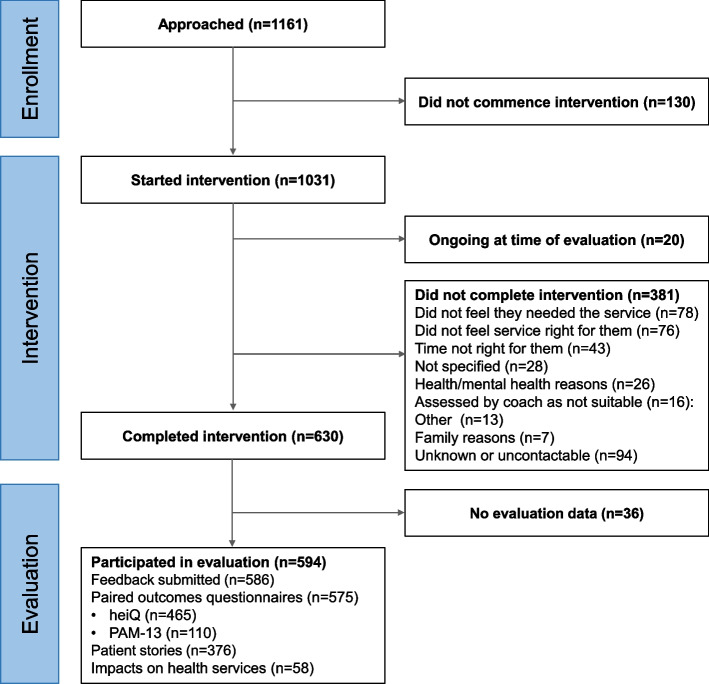
Participation flow chart

Participation is summarised in Fig. 2. A total of 1,161 patients were referred to the programme, 1,031 (88.8%) started, 381 (36.9% of starters) dropped out after commencing, and 20 did not complete within the evaluation timeframe. Of the 630 people who completed the intervention, 594 (94.2%) participated in the evaluation. Of the 594 participating in the evaluation, 586 (98.7%) returned feedback questionnaires, 376 (63.3%) gave permission for their anonymised stories to be shared, 479 (78.9%) were given heiQ questionnaires, and 115 (19.4%) were given PAM-13 questionnaires. Of those given heiQ questionnaires, 465 (97.1%) provided matched data. Of those given PAM-13 questionnaires, 110 (95.6% of 115) provided matched data. There were 208 missing data points (0.6% of 37,200) in the matched heiQ questionnaires and none in the matched PAM data (which allowed participants to choose ‘not applicable’).

### Participant characteristics

Participant characteristics are in Supplementary Table 1. Of the 630 who completed coaching, 390 (61.9%) provided demographic information, of whom 236 (60.5%) identified as female, and 347 (89.0%) as white British (reflecting local demographics). The most common age categories were 50-64y (31.5%) and 65-74y (29.5%). The average number of reported conditions per participant was 3.7. The most common condition category was depression/stress/anxiety, which affected 212 (54.4%) of the 390 completers with demographic data (a comorbidity for 196, 50.3%).

Of the 381 non-completers, 158 (41.5%) provided demographic information. Of these, 78 (49.4%) were female, 139 (88.0%) were white British, the most common age category was 65-74y (34.8%), the average number of conditions was 3.6 compared to 3.7 for completers, and 76 (48%) reported depression, stress, or anxiety.

### HeiQ results

Table 1 shows summary heiQ data alongside benchmark data from the Australian heiQ database [31]. An effect size between 0.2 and 0.5 is conventionally said to be 'small'; 0.5 to 0.8 is 'medium' and 0.8 or greater is 'large'. StACC outperformed benchmark effect-size data across each of the eight heiQ constructs.

**Table 1:** Summary heiQ data before and after the StACC intervention with benchmark comparisons (*n* = 465)

Construct	Timepoint	Mean	SD	Median	1st quartile (Q1)	3rd quartile (Q3)	StACC group effect size	Benchmark group effect size
**Health-directed Behaviour**	**Before**	2.67	0.74	2.75	2.25	3.25	0.51	0.37
	**After**	3.07	0.68	3.00	2.75	3.75		
	**Change**	0.32		0.25				
**Positive & Active Engagement in Life**	**Before**	2.84	0.62	2.80	2.40	3.20	0.62	0.35
	**After**	3.17	0.54	3.20	3.00	3.60		
	**Change**	0.32		0.20				
**Self-monitoring and Insight**	**Before**	2.96	0.41	3.00	2.67	3.17	0.54	0.34
	**After**	3.19	0.42	3.17	3.00	3.50		
	**Change**	0.23		0.17				
**Constructive Attitudes & Approaches**	**Before**	2.95	0.62	3.00	2.60	3.20	0.51	0.21
	**After**	3.22	0.55	3.20	3.00	3.60		
	**Change**	0.27		0.20				
**Skill and Technique Acquisition**	**Before**	2.65	0.52	2.75	2.25	3.00	0.66	0.43
	**After**	2.97	0.48	3.00	2.75	3.00		
	**Change**	0.32		0.25				
**Social Integration & Support**	**Before**	2.72	0.60	2.8	2.4	3.00	0.43	0.19
	**After**	2.97	0.59	3.00	2.60	3.40		
	**Change**	0.25		0.20				
**Health Services Navigation**	**Before**	2.94	0.51	3.00	2.60	3.20	0.36	0.19
	**After**	3.12	0.49	3.00	2.80	3.40		
	**Change**	0.17		0.20				
**Emotional Distress**	**Before**	2.52	0.67	2.50	2.00	3.00	-0.53	-0.21
	**After**	2.19	0.65	2.17	1.83	2.67		
	**Change**	-0.33		-0.33				

heiQ is scored between 1 (not yet realising their role in self-management) and 4 (consistent and effective self-managers) in each domain. The Emotional Distress domain should be negatively interpreted i.e. a negative effect is an improvement

### PAM-13 results

**Fig. 3:**
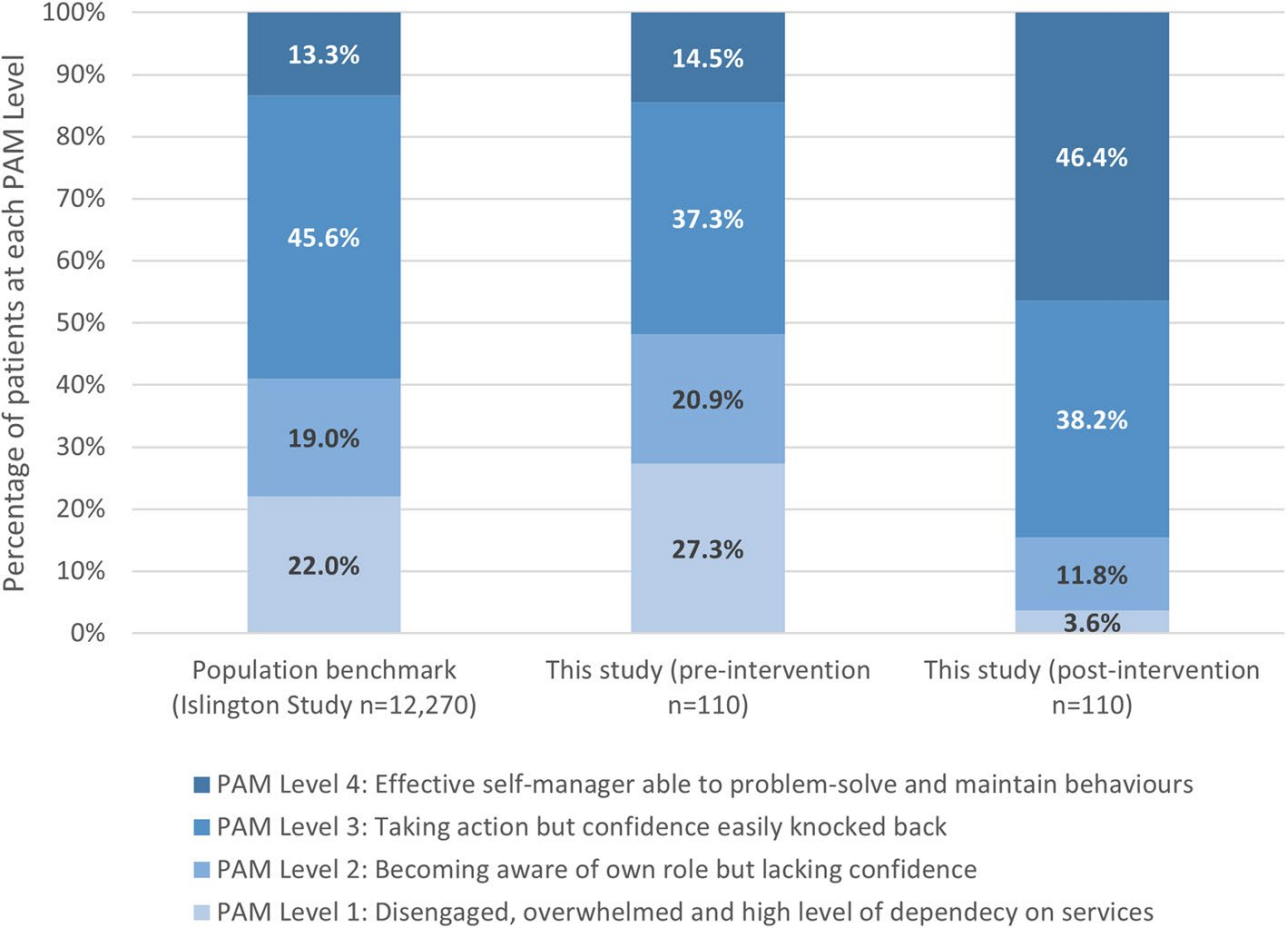
PAM levels pre and post intervention against population benchmark data. Legend: PAM levels for benchmark data (left), baseline for this study (centre) and after the StACC Intervention (right; *n* = 11). Benchmark data are from the Health Foundation Islington Study [7] where a PAM questionnaire was sent to 35,000 patients with long-term conditions and returned by 12,270 (35.1%)

Figure 3 shows the baseline and post-intervention PAM levels (centre and right) for all patients who completed matched PAM-13 surveys (*n* = 110) compared with population data (left) from 12,270 people with long-term conditions from the Health Foundation Islington Study [16]. Pre-intervention, there were *n* = 30 (27.3% of 110 participants) at the lowest PAM level (least engaged) and *n* = 16 (14.5%) at the highest PAM level (most engaged). Post-intervention, there were *n* = 4 (3.6%) at the lowest PAM level and *n* = 51 (46.4%) at the highest PAM level.

**Fig. 4:**
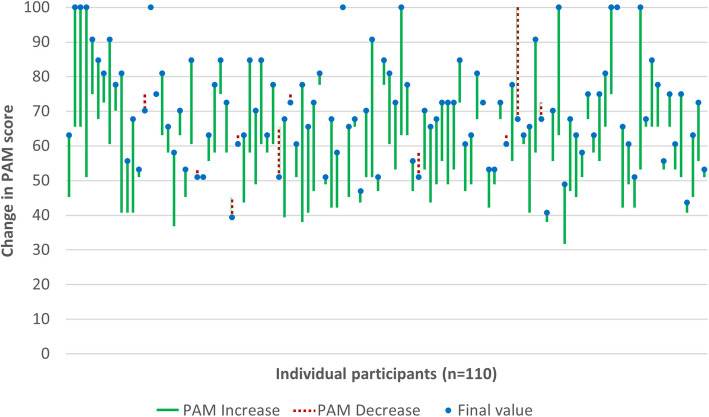
Individual changes in raw PAM score before and after the intervention (*n* = 110). Legend: The blue dot indicates the final score (T2). A green line indicates an increase. A red dotted line indicates a decrease. A dot with no line indicates a tie (T1 = T2)

Figure 4 shows the raw baseline and post-intervention PAM scores for each patient that completed both measures (*n* = 110). There were 95 increases (86.3%), 10 decreases (0.9%) and 5 ties (0.5%).

Table 2 shows that the pre-intervention mean PAM-13 score was 57.1 (SD = 13.6, 95% CI = 54.5–59.7), which increased to 70.8 (SD = 14.7, 95% CI = 68.1–73.6) post-intervention (*p* < 0.0001).

**Table 2:** Change in PAM-13 scores before and after the StACC intervention

Group	No	Pre-intervention			Post-intervention			Test	*P*-value
		**Mean**	**SD**	**95% conf. interval**	**Mean**	**SD**	**95% conf. interval**		
**Patient Activation Measures (PAM-13)**	110	57.1	13.6	[54.5–59.7]	70.8	14.7	[68.1–73.6]	Paired T-test	< 0.0001

### Health service usage

Monthly health service appointment data were provided in August 2017 for all 177 completers. After excluding people without 12 months post-intervention data (i.e. those completing after August 2016), there were 58 participants (9.2% of all completers). Health service usage pre- and post-intervention is presented in Table 3.

**Table 3:** Change in service usage 12 months before and after participants’ final StACC session (*n* = 58)

Resource	Total appts 12 m prior to end of coaching	Total appts 12 m following end of coaching	Change %	Pre-Intervention		Post-intervention		*p*-value
				Median appts per person per month	IQR	Median appts per person per month	IQR	
GP appts	555	448	-19.3	7	4–12	6	3–10	0.0225*
GP telephone appts	189	129	-31.7	3	0–4	1	0–3	0.0057*
GP home visits	18	8	-55.6	0	0–0	0	0–0	0.4521
Nurse appts	170	163	-4.1	1.5	0–4	2	0–4	0.9092
Healthcare assistant appts	191	175	-8.4	1.5	1–5	2	1–4	0.8366
Out of hours appts	7	3	-57.1	0	0–0	0	0–0	0.1772**
Hospital admissions	15	4	-73.3	0	0–0	0	0–0	0.1195**
Attendances at A&E	21	10	-52.4	0	0–0	0	0–0	0.1323**

Note to Table 3: *indicates significance at *p* < 0.05; **Home visits, out-of-hours appointments, hospital admissions and A&E attendances were insufficiently powered to exclude type II errors (inference of no effect from high *p*-values)

**Fig. 5:**
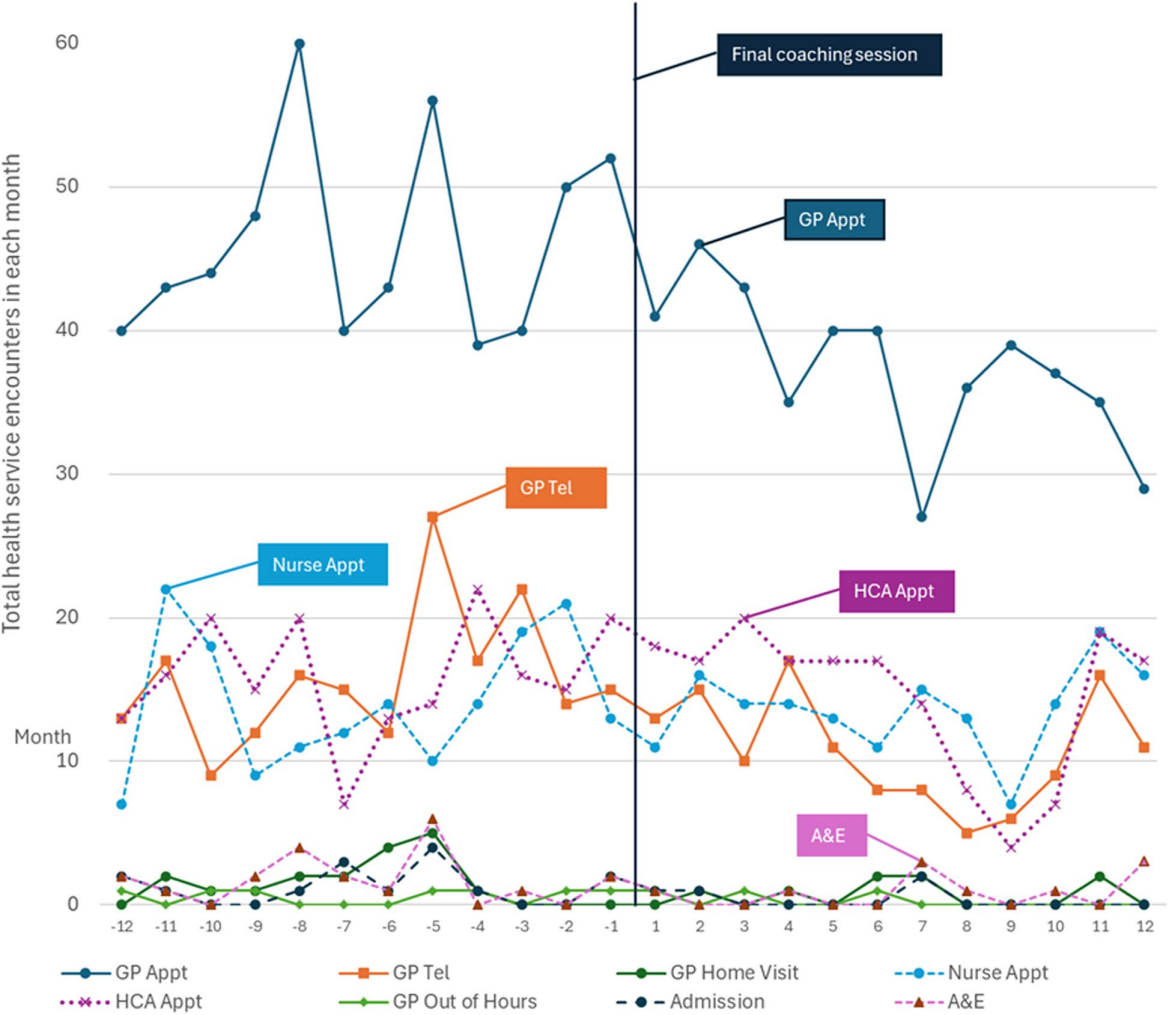
Monthly health service encounters 12 months before and after StACC intervention (*n* = 58). Legend: Appointment data was requested in August 2017. All participants for whom there were 12 months of complete GP records before and after the intervention were included. Secondary care data as recorded in GP records may be incomplete

Figure 5 shows monthly health service needs for all completers for whom there were 12 months of data before and after the final coaching session. Data were provided from GP records. Therefore, hospital records (A&E attendances, admissions) may be incomplete. There appears to be a significant drop in GP-led appointments, no drop in nurse-led or healthcare assistant-led appointments, and a possible drop in out-of-hours appointments, A&E attendances, and hospital admissions. Home visits, out-of-hours appointments, hospital admissions and A&E attendances were insufficiently powered to exclude type II errors (inference of no effect from high *p*-values). Given the small subgroup size, health service usage data should be interpreted as exploratory rather than confirmatory. Medical Research Council guidance is to report underpowered subgroup analyses “because they might be useful for subsequent meta-analyses, or for developing hypotheses for testing in further research” [31].

### Self-reported experiences and outcomes

A total of 586 feedback questionnaires were returned, of which 567 (96.8%) were positive (agree or strongly agree on a 4-point Likert scale) and 354 (60.4%) provided optional feedback comments. Neutral or negative responses related to the intervention not being needed, the patient wanting something else or, in two cases, being dissatisfied with the experience. A thematic summary of positive feedback comments generated themes relating to experiences, goals, problem-solving, confidence, actions and impacts (presented below). Each quote is from a different participant. Omissions are indicated by ellipses and clarifications by square brackets.

#### Experiences

Participants described their coaches as warm, understanding, empathetic, positive, but challenging. The coaching experience was described as an investment in their future, action-focused, driving transformative and lasting change that had helped them in different ways compared to other services. Participants described how important it was to be treated as a whole person with complex and interconnected physical, emotional and social needs.

*“Supportive and an excellent listener but gently assertive in helping me address my behaviours and look at [my problem]… She cleared the path and showed me the door was open.”*

#### Goals

Participants described how having the space to identify and reflect on what mattered to them supported them in setting and achieving their own goals, which, together with follow-up, were described as positive and motivating.

*“I felt very low and couldn't see how to live positively. [My coach] encouraged (and enabled) me to set goals, reach goals and view life positively. I no longer feel overwhelmed.”*

#### Problem-solving

Participants described new coping strategies, determination to address long-standing challenges, and transformative shifts in self-efficacy and approaches to problem-solving.

*“For me it has made me realise I was in a rut and has helped me realise that I could make a difference to how I controlled my health issues… it has reinforced that I can make a difference myself.”*

#### Confidence

Participants described a greater sense of control, increased self-esteem and confidence in making decisions, accessing information, coping with crises, and discussing and communicating their needs more openly.

*“The more that people know, the better it is for them. The service has provided confidence so better able to ask questions of medical professionals who have all been very good.”*

#### Actions

Reported actions included engaging with non-healthcare services, accessing peer support, addressing long-standing challenges, engaging with self-care, applying for work, and building new friendships.

*“…having the opportunity to discuss things around my health problems enabled me to make even more positive changes and to practise holistic self-care, as opposed to purely clinical self-care management and crisis prevention.”*

Participants described resolving bottleneck issues, resulting in broader benefits beyond the initial goal.

*“I thought the focus would be solely on my physical health, resolving the [work] issue above has had an impact on everything.”*

#### Impacts

Participants described stabilised symptoms, improved health behaviours, reduced risk factors, improved wellbeing, and reduced reliance on emergency healthcare. These helped them feel less overwhelmed about their condition(s) and more positive about their future.

*“The combination of this service and [mental health group] support have been so useful – I feel I have avoided hospital admissions because of it.”*

*“My blood sugars have come down, and I feel much more in charge of my health issues.”*

*“In terms of the speed and depth of positive changes, my time with my coach has been the most effective of my therapeutic experiences.”*

**Fig. 6:**
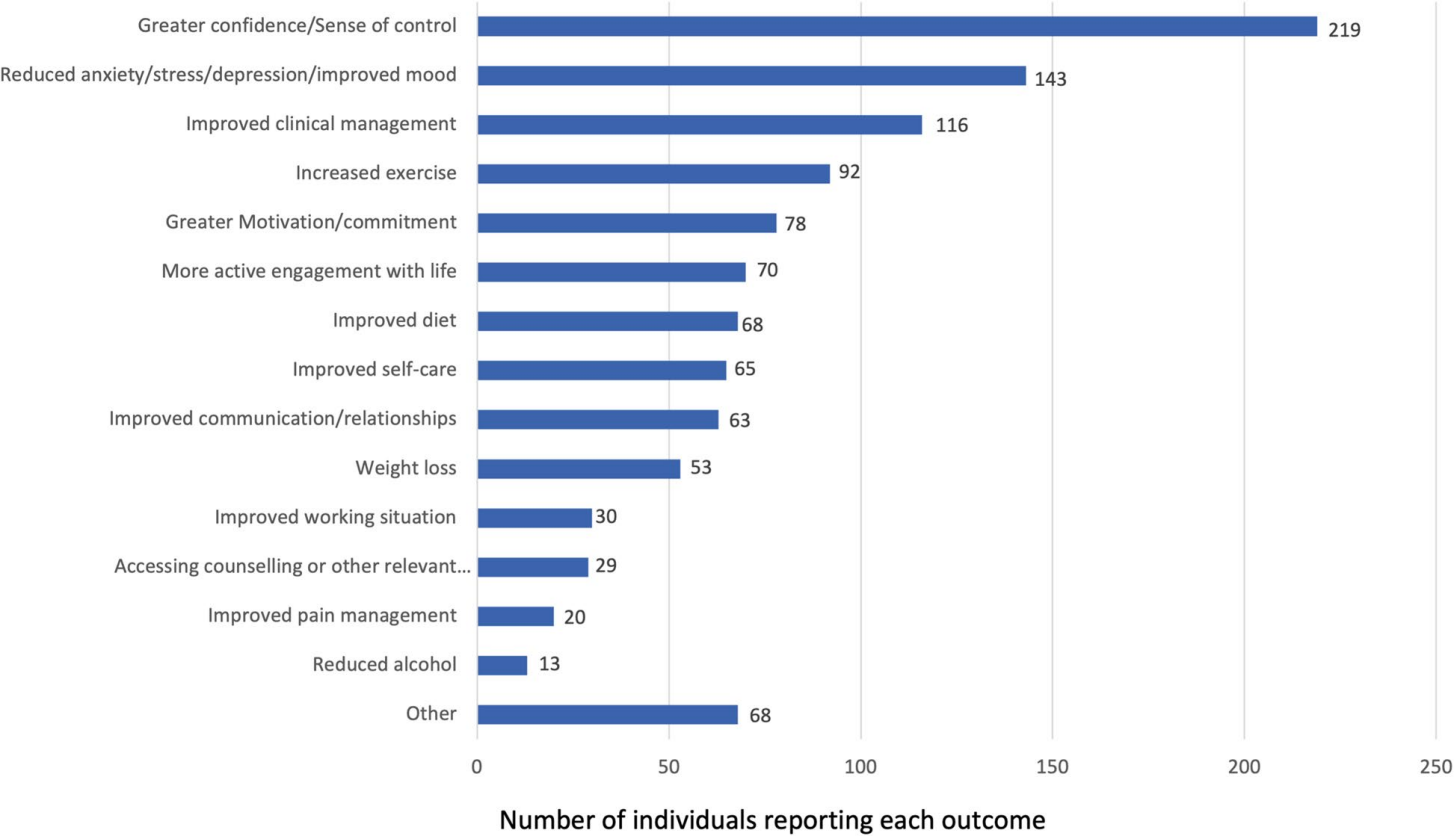
Self-described outcomes categorised according to theme (*n* = 376). Total adds up to more than 376 as some participants reported more than one outcome. Each participant was only counted once in each category, even if more than one outcome was reported in that category. Achieved goals or ongoing actions were counted but unactioned (intended-only) goals were not

A total of 376 completers (63.3%) consented to have their individual stories shared for insights into outcomes. Figure 6 shows self-reported impacts itemised, categorised, and charted according to frequency. Of these individual stories, 75 (19.9%) reported improvements under a single category, 90 (23.9%) under two categories, 90 (23.9%) under three categories, and 121 (32.2%) under four or more categories.

## Discussion

We have presented a mixed-methods evaluation of a health coaching intervention: the Structured Agenda-free Coaching Conversation (StACC) approach for people with long-term physical and mental health conditions and complex multimorbidity. This demonstrated above-benchmark improvements across all domains of the heiQ and significant improvements in patient activation as measured through PAM-13. These figures, combined with qualitatively reported outcomes, suggest that health coaching conversations are an effective intervention in this group. Previous research indicates that higher heiQ scores correlate with improved self-management skills, health behaviours, attitudes and feelings [25] and that higher PAM scores correlate with improved health outcomes and reduced acute healthcare usage [32]. Our findings support these studies.

Our qualitative analysis suggests that people who were previously disengaged or overwhelmed can be supported through coaching conversations to take ownership, tap into their own motivation, draw on their own strengths, and build their confidence and problem-solving skills. Once ‘activated’, people are more likely to engage in self-management actions, positively impacting their health and wellbeing and reducing their reliance on acute health services.

The wide range of self-described outcomes from patient stories (Fig. 6) supports the potential applicability of this approach to multimorbidity and non-health-related issues, which require personalised approaches [33]. Many participants reported multiple outcomes, and the subgroup analysis of health-service needs suggested a sustained effect. This supports our inference that participants developed new problem-solving skills and applied them to issues beyond the primary goal discussed within their coaching sessions.

The applicability of StACC to diverse health conditions and problems, and its delivery by non-clinical coaches, has health service implications. Internationally, many healthcare systems are struggling to meet the needs of rising numbers of people with long-term health conditions and multimorbidity in ways that are equitable and socially just [34]. Its replicability across seven primary care sites makes StACC a promising model for broader implementation.

The scale of the change to health service usage suggested by our small subgroup analysis aligns with the data collected in the Health Foundation’s Islington Study [32] which found that the difference in need for GP appointments for patients at activation level 1 and level 4 was 19%. Although health service usage has only been quantitatively investigated for a small subgroup, qualitatively reported outcomes further support an inference of reduced reliance on clinical services. It is also notable that although GP encounters showed a significant decrease, there was no associated reduction in nurse-led or healthcare assistant-led appointments. This would be consistent with engaged self-management, as these professionals tend to lead proactive care-planning appointments. Both hospital admissions and A&E appointments appeared to show an absolute drop. However, this study was not sufficiently powered to determine the significance of this drop.

There appeared to be a higher proportion of men, older people, and people from minoritised ethnic backgrounds who did not complete the intervention. There is some uncertainty (particularly about ethnicity) as the absolute numbers were low. However, this would be consistent with other research indicating barriers to talking interventions for these groups [35].

Notably, people referred to this coaching program had lower initial activation levels (more people starting at levels 1 and 2) compared to the benchmarks from the Health Foundation’s Islington Study for people with long-term conditions [32], dispelling initial concerns that only highly 'activated' patients would respond and engage. The higher proportion of participants with depression, anxiety or stress in the completers group, compared to non-completers, also implies that people in this category valued the intervention. StACC consists of five or six coaching sessions, which have cost implications for providers and time implications for patients. Positive patient feedback indicates that those completing the intervention found the time commitment acceptable. Further research is needed to explore cost-effectiveness and cost benefits.

### Limitations

The main limitation of this study is that it is an evaluation, therefore without sampling, controls or randomisation. We argue, however, that evaluative studies are warranted given the known challenges for quasi-experimental research relating to multimorbidity and personalised care [33].

Another key limitation is the potential for bias, given that the coaches delivering the intervention were involved in data collection, and one author (KH) was the service lead. Two independent health service researchers (KLG and AO) who audited the data and were involved in its interpretation helped to mitigate this. Differential attrition is also likely to affect feedback, as only those completing the intervention provided it.

The entire cohort was not observed through a single measure. However, each different type of evaluation (PAM-13, heiQ, service utilisation, and qualitative feedback) points towards similarly positive outcomes. As cautioned in the results, the impacts on health service usage are for a small subset. The implied effect size, however, may support power calculations for future researchers.

Benchmark heiQ data may not be comparable to UK self-management programmes, as they were from the Australian heiQ database where there is a hybrid public–private healthcare system [36]. This intervention was evaluated across seven UK primary care sites before the COVID-19 pandemic. Further studies are needed to confirm transferability to different contexts.

## Conclusion

This evaluation suggests that the StACC intervention improved patient activation PAM-13 scores, heiQ scores, and self-reported patient outcomes. Improvements in these are associated with reduced reliance on acute health services, an inference supported by a subgroup analysis. Follow-up efficacy and economic studies are warranted, as is an exploration of transferability to other contexts.

## Supplementary Information

Supplementary Material 1.

## Data Availability

The datasets used and/or analysed during the current study are available from the corresponding author on reasonable request.

## Abbreviations

GP: General Practice
heiQ: Health Education Impact Questionnaire
NHS: National Health Service
PAM: Patient Activation Measure
PAM-13: Patient Activation Measure-13 item instrument
StACC: The Structured Agenda-free Coaching Conversation
T-GROW: Topic, Goal, Reality, Options, Will/Way forward (a coaching model)

## References

[1] Wagner EH. Chronic disease management: what will it take to improve care for chronic illness? Eff Clin Pract. 1998;1(1):2–4. 10345255

[2] Wanless D. Securing our Future Health: Taking a Long-Term View. UK Treasury; 2022.

[3] Tattersall RL. The expert patient: a new approach to chronic disease management for the twenty-first century. Clin Med (Lond). 2002;2(3):227–9. 12108472 10.7861/clinmedicine.2-3-227 PMC4954037

[4] Resnicow K, McMaster F. Motivational Interviewing: moving from why to how with autonomy support. Int J Behav Nutr Phys Act. 2012;9:19. 22385702 10.1186/1479-5868-9-19 PMC3330017

[5] Foundation TH. Evidence: Helping people help themselves. 2011.

[6] El-Osta A, Webber D, Gnani S, Banarsee R, Mummery D, Majeed A, et al. The Self-Care Matrix: a unifying framework for self-care. Self-Care. 2019;10:38–56.

[7] Wagner EH, Austin BT, Von Korff M. Organizing care for patients with chronic illness. Milbank Q. 1996. 10.2307/3350391. 8941260

[8] Coulter A, Kramer G, Warren T, Salisbury C. Building the House of Care for people with long-term conditions: the foundation of the House of Care framework. Br J Gen Pract. 2016;66(645):e288–90. 27033503 10.3399/bjgp16X684745 PMC4809714

[9] Chambers E, Coleman K. Enablers and barriers for engaged, informed individuals and carers: left wall of the House of Care framework. Br J Gen Pract. 2016;66(643):108–9. 26823262 10.3399/bjgp16X683797 PMC4723188

[10] All Party Parliamentary Group on Primary Care & Public Health. Ten years on from Wanless, how “fully-engaged” are we? ; 2012 17th April.

[11] Downey M. Effective Coaching: Lessons from the Coaches' Coach: Texere; 2003.

[12] Johnston MC, Crilly M, Black C, Prescott GJ, Mercer SW. Defining and measuring multimorbidity: a systematic review of systematic reviews. Eur J Public Health. 2019;29(1):182–9. 29878097 10.1093/eurpub/cky098

[13] Hibbard JH, Mahoney ER, Stock R, Tusler M. Do increases in patient activation result in improved self-management behaviors? Health Serv Res. 2007;42(4):1443–63. 17610432 10.1111/j.1475-6773.2006.00669.x PMC1955271

[14] Mirmazhari R, Ghafourifard M, Sheikhalipour Z. Relationship between patient activation and self-efficacy among patients undergoing hemodialysis: a cross-sectional study. Renal Replacement Therapy. 2022;8(1):40.

[15] Greene J, Hibbard JH, Sacks R, Overton V, Parrotta CD. When patient activation levels change, health outcomes and costs change, too. Health Aff (Millwood). 2015;34(3):431–7. 25732493 10.1377/hlthaff.2014.0452

[16] Deeny S, Thorlby R, Steventon A. Reducing emergency admissions. 2018.

[17] Greene J, Hibbard JH. Why does patient activation matter? An examination of the relationships between patient activation and health-related outcomes. J Gen Intern Med. 2012;27(5):520–6. 22127797 10.1007/s11606-011-1931-2 PMC3326094

[18] Hibbard JH, Greene J. What the evidence shows about patient activation: better health outcomes and care experiences; fewer data on costs. Health Aff (Millwood). 2013;32(2):207–14. 23381511 10.1377/hlthaff.2012.1061

[19] Wallace LM, Turner A, Kosmala-Anderson J, Sharma S, Jesuthasan J, Bourne CL, et al., editors. Co-creating health : evaluation of first phase2012.

[20] Newbronner L, Foundation H, Staff HF, Research F, Evaluation, Research F, et al. Sustaining and Spreading Self-Management Support: Lessons from Co-Creating Health Phase 2: Health Foundation; 2013.

[21] Lorig KR, Sobel DS, Ritter PL, Laurent D, Hobbs M. Effect of a self-management program on patients with chronic disease. Eff Clin Pract. 2001;4(6):256–62. 11769298

[22] Sobel DS, Lorig K, Hobbs M, editors. Chronic Disease Self-Management Program: From Development to Dissemination2002.

[23] Miller WR, Rollnick S. Motivational Interviewing. Preparing People for Change. New York, NY: The Guilford Press; 2002.

[24] Health Research Authority. Defining Research 2022. Available from: https://www.hra-decisiontools.org.uk/research/docs/DefiningResearchTable_Oct2022.pdf.

[25] Osborne RH, Elsworth GR, Whitfield K. The health education impact questionnaire (heiQ): an outcomes and evaluation measure for patient education and self-management interventions for people with chronic conditions. Patient Educ Couns. 2007;66(2):192–201. 17320338 10.1016/j.pec.2006.12.002

[26] Hibbard JH, Stockard J, Mahoney ER, Tusler M. Development of the patient activation measure (PAM): conceptualizing and measuring activation in patients and consumers. Health Serv Res. 2004;39(4 Pt 1):1005–26. 15230939 10.1111/j.1475-6773.2004.00269.x PMC1361049

[27] Patient Activation Measure (PAM): Insignia Health; Available from: https://www.insigniahealth.com/pam/.

[28] Ng QX, Liau MYQ, Tan YY, Tang ASP, Ong C, Thumboo J, et al. A Systematic Review of the Reliability and Validity of the Patient Activation Measure Tool. Healthcare. 2024;12(11):1079. 38891154 10.3390/healthcare12111079 PMC11171848

[29] Bachrach C, Newcomer SF. Addressing bias in intervention research: summary of a workshop. J Adolesc Health. 2002;31(4):311–21. 12359376 10.1016/s1054-139x(02)00395-6

[30] Braun V, Clarke V. Thematic analysis. APA handbook of research methods in psychology, Vol 2: Research designs: Quantitative, qualitative, neuropsychological, and biological. APA handbooks in psychology®. Washington, DC, US: American Psychological Association; 2012. p. 57–71.

[31] Elsworth GR, Osborne RH. Percentile ranks and benchmark estimates of change for the Health Education Impact Questionnaire: normative data from an Australian sample. SAGE Open Med. 2017;5: 2050312117695716. 28560039 10.1177/2050312117695716 PMC5435365

[32] Barker I, Steventon A, Williamson R, Deeny SR. Self-management capability in patients with long-term conditions is associated with reduced healthcare utilisation across a whole health economy: cross-sectional analysis of electronic health records. BMJ Qual Saf. 2018;27(12):989–99. 30139822 10.1136/bmjqs-2017-007635 PMC6288702

[33] Nimmons D, Hyde E, Leedham-Green K. Addressing multimorbidity through personalised care. In: Leedham-Green K, Park S, editors. Generalism in Clinical Practice and Education: UCL Press; 2024. p. 397–426.

[34] Mercer S, Gillies J, MacRae C. Organisation and design of healthcare for generalism. In: Park S, Leedham-Green K, editors. Generalism in Clinical Practice and Education: UCL Press; 2024. p. 167–92.

[35] Sharland E, Rzepnicka K, Schneider D, Finning K, Pawelek P, Saunders R, et al. Socio-demographic differences in access to psychological treatment services: evidence from a national cohort study. Psychol Med. 2023;53(15):7395–406. 37194490 10.1017/S0033291723001010 PMC10721408

[36] Cancarevic I, Plichtová L, Malik BH. Healthcare Systems Around the World. In: Tohid H, Maibach H, editors. International Medical Graduates in the United States: A Complete Guide to Challenges and Solutions. Cham: Springer International Publishing; 2021. p. 45–79.

