# Supplementary material for: Health coaching for people with long-term conditions and multimorbidity: a mixed methods prospective service evaluation of Structured Agenda-free Coaching Conversations (StACC) in UK primary care
Source: BMC Public Health. 2025 Jul 3;25:2365. doi: 10.1186/s12889-025-23535-0 (PMC12225140; doi:10.1186/s12889-025-23535-0)
Supplement: Supplementary file 1 — Supplementary Material 1. [file 12889_2025_23535_MOESM1_ESM.docx]

# **Supplemental Table 1**

# **What are the impacts of a Structured Agenda-free Coaching Conversation approach (StACC) on patient activation, health service needs and health outcomes for people with long-term health conditions and multimorbidity? A prospective service evaluation in UK primary care**

### Supplemental Table 1: Participant characteristics

|  | **All (n=548)** | | **Completers (n=390)** | |
| --- | --- | --- | --- | --- |
|  | **Frequency** | **Percentage** | **Frequency** | **Percentage** |
| **Gender** |  |  |  |  |
| Female | 314 | 57.3% | 236 | 61.0% |
| Male | 224 | 40.9% | 148 | 38.0% |
| Missing | 10 | 1.8% | 6 | 1.0% |
| **Age** |  |  |  |  |
| 18-49 | 80 | 14.6% | 58 | 14.9% |
| 50-64 | 162 | 29.6% | 123 | 31.5% |
| 65-74 | 170 | 31.0% | 115 | 29.5% |
| 75-84 | 92 | 16.8% | 65 | 16.7% |
| 85+ | 22 | 4.0% | 14 | 3.6% |
| Missing | 22 | 4.0% | 15 | 3.8% |
| **Ethnicity** |  |  |  |  |
| White British | 486 | 88.7% | 347 | 89.0% |
| White other | 30 | 5.5% | 23 | 5.9% |
| Mixed/multiple ethnicity | 6 | 1.1% | 5 | 1.3% |
| Asian/Asian British | 10 | 1.8% | 3 | 0.8% |
| Black/African/Caribbean | 2 | 0.4% | 1 | 0.3% |
| Other (please specify) | 2 | 0.4% | 2 | 0.4% |
| Missing | 12 | 2.2% | 9 | 2.3% |
| **Current health conditions*** |  |  |  |  |
| Osteoarthritis | 150 | 27.4% | 110 | 28.2% |
| Rheumatoid Arthritis | 59 | 10.8% | 48 | 12.3% |
| Respiratory Conditions | 175 | 31.9% | 121 | 31.0% |
| Heart Condition | 125 | 22.8% | 81 | 20.8% |
| Stroke | 45 | 8.2% | 30 | 7.7% |
| Diabetes | 165 | 30.1% | 108 | 27.7% |
| Blood Pressure | 260 | 47.4% | 187 | 47.9% |
| Neurological Disorder | 42 | 7.7% | 32 | 8.2% |
| Gastric Condition | 147 | 26.8% | 101 | 25.9% |
| Multiple Sclerosis | 2 | 0.4% | 0 | 0.0% |
| Chronic Fatigue Syndrome | 34 | 6.2% | 29 | 7.4% |
| Depression/Stress/Anxiety | 288 | 52.6% | 212 | 54.4% |
| Other | 500 | 91.2% | 364 | 93.3% |

*Conditions add up to more than 100% as participants could choose more than one
